# INFLUENCE OF GAIT-SYNCHRONIZED FUNCTIONAL ELECTRICAL STIMULATION DURING EXOSKELETON-ASSISTED AMBULATION ON CARDIORESPIRATORY OUTCOMES IN INDIVIDUALS WITH INCOMPLETE SPINAL CORD INJURY

**DOI:** 10.2340/jrm.v57.43423

**Published:** 2025-11-02

**Authors:** Robert VOICU, Daniela B. KUCHEN, Claudio PERRET, Ines BERSCH, Mario WIDMER

**Affiliations:** 1 Neuro-Musculoskeletal Functioning and Mobility Group, Swiss Paraplegic Research, Nottwil; 2 Department of Health Sciences and Technology, ETH Zurich, Zurich; 3 Department of Therapy, Swiss Paraplegic Centre, Nottwil; 4 Institute for Social and Preventive Medicine, University of Bern, Bern; 5 Faculty of Health Sciences and Medicine, University of Lucerne, Lucerne; 6 International FES Centre®, Swiss Paraplegic Centre, Nottwil, Switzerland

**Keywords:** spinal cord injuries, electric stimulation therapy, exoskeleton device, gait, neurological rehabilitation, cardiorespiratory fitness, exercise test, crossover studies

## Abstract

**Objective:**

To evaluate the impact of gait-synchronized functional electrical stimulation during exoskeleton-assisted ambulation on cardiorespiratory demand in individuals with incomplete spinal cord injury.

**Design:**

Cross-sectional study employing a randomized crossover arrangement of measurements.

**Participants:**

Convenience sample of 11 individuals with chronic incomplete spinal cord injury and partial walking ability.

**Methods:**

Participants completed 2 x 6-min walking tests (6MWTs) with the EksoNR, 1 with and 1 with-out gait-synchronized functional electrical stimulation targeting gait-related muscles in a randomized order. Cardiorespiratory and metabolic parameters were measured breath-by-breath via ergospirometry. The primary outcome was oxygen consumption (V̇O_2_/kg). Secondary outcomes included further cardiovascular and metabolic parameters. Data from the final 2 min of each 6MWT were analysed using linear mixed-effect models.

**Results:**

V̇O_2_/kg increased by 6% with functional electrical stimulation compared with exoskeleton-assisted ambulation alone (15.07 ± 4.11 vs 14.21 ± 3.61 mL·min^-^¹·kg^-^¹, *p* = 0.02). Heart rate, ventilation, and energy expenditure were also elevated (*p* < 0.05), while breathing frequency, respiratory exchange ratio, distance, and perceived exertion remained unchanged. Inter-individual differences in V̇O_2_/kg were not explained by stimulation amplitude (*r* = 0.36, *p* = 0.27).

**Conclusion:**

Adding functional electrical stimulation to exoskeleton-assisted gait therapy consistently increased cardiorespiratory demand, potentially enhancing training intensity. Further research assessing long-term clinical impact is required.

## LAY ABSTRACT

People with incomplete spinal cord injury often experience reduced mobility and fitness. This study tested whether using functional electrical muscle stimulation while walking in a robotic exoskeleton would increase physical effort compared with walking with the exoskeleton without stimulation. Therefore, 11 participants walked with and without muscle stimulation using the exoskeleton. The results show that adding functional electrical stimulation while walking with a robotic exoskeleton significantly increased the amount of oxygen consumed by the body, indicating higher muscle activity. On average, oxygen consumption was increased by 6%. Heart rate and breathing also showed increases, suggesting a more intense workout. These findings are important because activating muscles more during rehabilitation may enhance gait function, weight management, and overall health in people with incomplete spinal cord injury. Our results support the use of exoskeleton therapy in combination with electrical muscle stimulation, but further research needs to evaluate the long-term impact.

In spinal cord injury (SCI), disruption of the communication pathways between the brain and the body leads to partial or complete loss of motor, sensory, and autonomic functions below the injury level. Depending on the extent and location, the injury can be classified as either complete or incomplete spinal cord injury (1). Both complete and incomplete SCI negatively affect motor function as well as cardiovascular and respiratory fitness, often resulting in reduced maximal oxygen uptake and aerobic capacity (2–4). This decline is primarily due to decreased physical activity, muscle atrophy, and deconditioning of the cardiovascular and respiratory systems (5). Additionally, autonomic nervous system function is affected, contributing to impaired heart rate and blood pressure regulation, weakened respiratory muscles, and diminished lung capacity (6). Together, these challenges increase the risk of secondary complications such as cardiovascular disease, metabolic conditions, respiratory dysfunction, and weight gain, all of which negatively affect rehabilitation and general quality of life (7).

In individuals with incomplete SCI, residual communication pathways between the brain and the body parts below the lesion allow some functional recovery through neuroplasticity, making rehabilitation an important priority (8). Regaining gait, balance, and mobility are among the most important goals for rehabilitation after incomplete SCI (9). While conventional rehabilitation for incomplete SCI focuses on physiotherapy and the use of assistive devices, these approaches are often limited in their ability in addressing gait function and cardiovascular fitness (10). Innovations in rehabilitation technology, such as powered exoskeletons with synchronized functional electrical stimulation (FES), have emerged as promising modalities for enhancing rehabilitation outcomes. Powered exoskeletons are wearable robotic devices that facilitate repetitive, task-specific training promoting neural recovery and muscle re-education (11). Studies indicate that exoskeleton-assisted therapy is a feasible and safe intervention for incomplete SCI patients and has demonstrated beneficial effects on gait function, balance, and mobility in these patients (9). However, the efficacy of that approach is limited by the difficulty of recruiting muscle fibres below the level of injury (12). FES is a rehabilitation technique that applies electrical currents to activate nerves innervating extremities affected by paralysis (13). FES has been shown to prevent disuse atrophy, improve muscle strength, power output, endurance, and cardiovascular fitness, and reduce body fat mass (14–22). Moreover, Hettinga and Andrews (21) have shown that combining FES with volitional exercise leads to a significantly higher oxygen consumption compared with the same activity without FES.

Both exoskeleton therapy and FES have independently been shown to support physical rehabilitation. However, a combined approach of both therapies has the potential for synergistic effects, mainly by increasing training intensity, enhancing muscle fibre recruitment, improving cardiovascular fitness, and ultimately leading to higher calorie consumption (23–25). Despite promising theoretical benefits, there is currently limited evidence to support their combined use in clinical practice (26).

We examined whether combining gait training using a powered exoskeleton with gait-synchronized FES had an influence on cardiorespiratory demand compared with using a powered exoskeleton alone. It was hypothesized that oxygen consumption would increase in the combined therapy due to the increased number of recruited muscle fibres induced by gait-synchronized FES.

## METHODS

### Study design and setting

This cross-sectional study using a randomized crossover arrangement of measurements was a subproject of an ongoing monocentric, randomized clinical trial (RCT) performed at the Swiss Paraplegic Centre (SPC) in Nottwil, Switzerland.

The study protocol was reviewed and approved by the responsible ethics committee Ethikkommission Nordwest- und Zentralschweiz (BASEC ID: 2021-D0039). All subjects gave written informed consent in accordance with the Declaration of Helsinki. The trial was registered at clinicaltrials.gov (ID: NCT05187650).

### Study population

Eligible participants were adults (≥ 18 years) with a chronic incomplete SCI (> 1 year) classified as American Spinal Injury Association (ASIA) Impairment Scale (AIS) B–D, capable of performing a 10-m walk test (10MWT) by themselves with or without walking aids but with partial wheelchair-dependence in everyday life. An intact lower motoneuron at the segmental innervation level of the gluteal muscles, hamstrings, and quadriceps muscles was essential to ensure compatibility with FES.

Exclusion criteria were based on device-specific considerations such as bodyweight exceeding 100 kg, height under/over 155/190 cm (unless they have a thigh length within 51.0 cm to 61.4 cm and a shank length within 48.0 cm–63.4 cm), and pelvic width > 46 cm. Further exclusion criteria were fractures, contractures, heterotopic ossification, severe spasticity (Modified Ashworth Scale > 3 in every key muscle), skin injuries on the lower limbs, unstable circulation, acute deep vein thrombosis, or pregnancy. Finally, participants with significant medical comorbidities or concurrent neurological injuries were excluded.

### Procedure

**Fig. 1:**
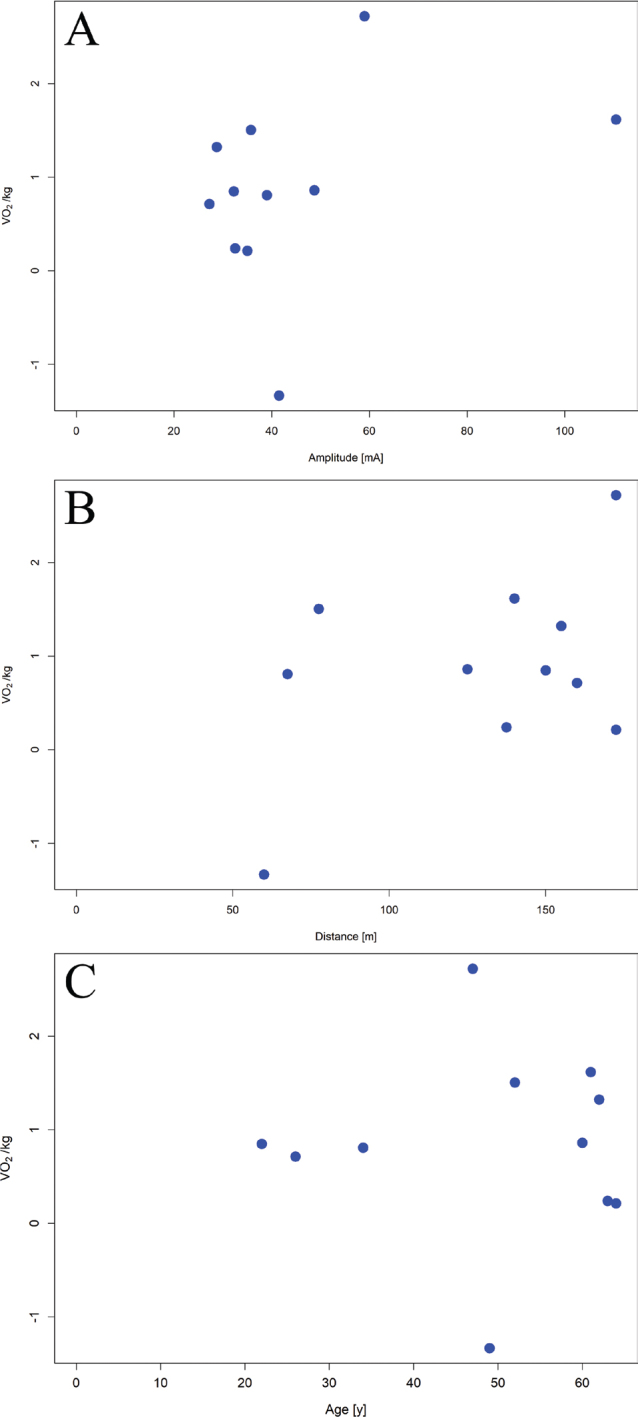
Overview of the study process featuring a randomized crossover design after a resting and a familiarization measurement. R refers to randomization of the order of conditions.

Fig. 1 illustrates the study procedure and the participant flow through the study. Participants in this sub-project were recruited from the list of participants in the main project already mentioned. Measurements for each participant were conducted during a single visit. After obtaining informed consent and verifying in- and exclusion criteria, the baseline metabolic data for each participant at rest were collected using the wearable K5 device (K5 wearable metabolic system, COSMED, Rome, Italy). Afterwards, a pair of self-adhesive electrodes (Axelgaard Pals, Lystrup, Denmark) were applied bilaterally to each of the targeted muscle groups (gluteal muscles, hamstrings, quadriceps, and gastrocnemius). Gait-synchronized FES was applied using the RehaMove2 stimulator (Hasomed, Magdeburg, Germany). FES amplitudes were individually adapted based on the participant’s remaining motor and sensory function to the highest level they could tolerate. The remaining FES parameters were standardized across all measurements in terms of frequency (35 Hz), pulse width (300 μs) and a ramp-up and -down time of 1 s.

Subsequently, the participant was transferred to the EksoNR exoskeleton (Ekso Bionics, Richmond, CA, USA) and a 6MWT without cardiometabolic measurement was carried out to allow familiarization. This phase was intended to help the participant acclimatize to the device, to familiarize with walking, and to get used to walking for 6 min with the EksoNR combined with FES. Meanwhile, therapists adjusted the EksoNR device settings, including step length, height, and the required level of device assistance. Participants used the crutches included with the system for additional support while walking. The EksoNR allows participants to walk in 2 different modes when utilized in conjunction with FES: the adaptive and the maximum mode. The mode was determined according to the participant’s residual gait function. In adaptive mode, the device provides assistance only as needed, detecting the user’s step initiation and supporting the swing trajectory to promote gait symmetry. In maximum mode, the exoskeleton delivers full support for gait movements irrespective of voluntary input. In both modes, the therapist can set parameters such as step length, step height, and gait speed. Following mode selection and initial adjustments, exoskeleton parameters were fixed, FES amplitudes were verified, and these settings were retained for the rest of the protocol.

After a 15-min rest, participants were randomly assigned to either perform the 6MWT using the EksoNR with FES first, followed by EksoNR without FES, or vice versa. FES was synchronized with the EksoNR, stimulating key muscles (gluteal muscles, hamstrings, quadriceps, and gastrocnemius) based on the phase of gait (27). Between the 2 x 6MWTs, a 15-min break in a seated position was taken, to allow breathing and heart rate (HR) to return to a resting state, as confirmed by a spiroergometry measurement during the last 5 min of the break. Participants were instructed to walk at a self-selected, comfortable pace. During each 6MWT, respiratory gases were measured, with data from the last 2 min used for analysis to account for stabilization of metabolic variables. After each 6MWT, the total distance covered was recorded, and participants were asked to report their rate of perceived exertion (RPE). RPE was evaluated using the Borg scale, ranging from 6 (no exertion at all) to 20 (maximal exertion) (28).

Finally, safety assessments were conducted on skin contact areas to identify any signs of irritation or potential complications.

### Variables

Cardiorespiratory and metabolic parameters were measured breath-by-breath using the wearable K5 device, which was freshly calibrated before each measurement. The primary outcome was the within-subject difference of cardiorespiratory demand between walking with EksoNR with and without FES measured as oxygen consumption per kilogram body mass (V̇O2/kg). Secondary outcomes included complementary cardiovascular and metabolic parameters such as minute ventilation (V̇E), HR, breathing frequency (BF), respiratory exchange ratio (RER), energy expenditure (EE), distance, and RPE.

### Sample size

*A priori* sample size estimation was conducted to ensure adequate statistical power for detecting within-subject differences in cardiorespiratory demand. As the investigated response was a direct physiological effect and not a training effect that needs to be converted into a clinical outcome measure, we assumed a large effect size. A large effect size of 1 could be detected with 80% statistical power at a significance level of 5% using a sample size of 11 participants.

### Data analysis

Descriptive data analysis was conducted to summarize the study findings, with results presented in tables, boxplots, and text as appropriate. Data management, statistical analyses, and visualization were performed using R Studio (Version 4.4.1; R Foundation for Statistical Computing, Vienna, Austria) and Microsoft Excel (Microsoft Office Professional Plus 2019; Microsoft Corp, Redmond, WA, USA).

For our primary analysis, we used a linear mixed-effect model (LMM) implemented in the lme4 package in R (29). Condition (Ekso, EksoFES; reference: Ekso) and sequence (Ekso-EksoFES, EksoFES-Ekso; reference: Ekso-EksoFES) were considered as fixed effects. A subject-specific random intercept accounted for within-subject correlation. This model structure estimates the change in V̇O_2_/kg for the EksoFES condition compared with Ekso without FES. Mixed model estimate (β), standard error (SE), and *p*-values were reported to determine significance as a 2-sided threshold of 0.05. Likelihood ratio test (LRT) was used to evaluate whether the sequence influences the outcome.

Secondary outcomes were also analysed using LMMs, adhering to the same framework and model structure described earlier. Moreover, Pearson correlations were used to explore relationships between the change in V̇O_2_/kg from Ekso to EksoFES and the average stimulation amplitude of all key muscles, the average distance walked, and the age of the participants.

For all the LMMs described, normality of residuals was confirmed by the Shapiro–Wilk test**.** The order of conditions (i.e., Ekso-EksoFES or EksoFES-Ekso) was randomized using a custom R program to reduce potential order effects.

## RESULTS

### Participants

All 11 participants were recruited between August 2024 and November 2024. Table I summarizes characteristics of the convenience sample and Fig. 1 illustrates the participant flow through the study. The neurological level of injury (NLI) and AIS scores ranged from C3 to L2, AIS B–D. Ten participants walked using the adaptive mode of the exoskeleton, whereas only 1 used the maximum mode due to limited residual walking function. Except for 1, all participants had prior experience with the exoskeleton. Additionally, all participants were familiar with FES, from either home use or participation in other studies. Seven participants had experience specifically with the combination of the exoskeleton and FES, with their last exposure ranging from 0 to 30 months ago.

**Table I:** Characteristics of the study population

Characteristics	
*n*	11
Age (years), mean ± SD	49.1 ± 15.3
Sex, *n* (%)	
Male	9 (82)
Female	2 (18)
Height (cm), mean ± SD	168.5 ± 25.0
Weight (kg), mean ± SD	82.9 ± 11.5
BMI (kg·m⁻²), mean ± SD	26.6 ± 3.1
Neurological level of injury (NLI) and severity of injury, *n* (%)	
C1–C4 AIS A, B, C	0 (0)
C5–C8 AIS A, B, C	0 (0)
T1–S5 AIS A, B, C	2 (18)
All AIS D	9 (82)
Type of injury, *n* (%)	
Traumatic	6 (55)
Non-traumatic	5 (45)
Time since injury (years), mean ± SD	6.5 ± 6.9
Comorbidities, *n* (%)	
History of cardiovascular disease	5 (45)
History of lung diseases	3 (27)
Baseline walking ability (without exoskeleton)	
10MWT, self-selected (m/s), mean ± SD	0.62 ± 0.31
10MWT, maximal (m/s), mean ± SD	0.82 ± 0.44
6MWT (m), mean ± SD	221 ± 111
WISCI II [0–20], median (range)	16 (9–16)

“All AIS D” includes participants with NLI from C3 to L2; AIS A–C are shown by NLI ranges according to the International SCI Core Data Set. AIS: American Spinal Injury Association Impairment Scale; C: cervical; T: thoracic; S: sacral; SD: standard deviation, 10MWT: 10-m walk test, 6MWT: 6-min walking test, WISCI II: Walking Index for Spinal Cord Injury II.

### Primary outcome

**Fig. 2:**
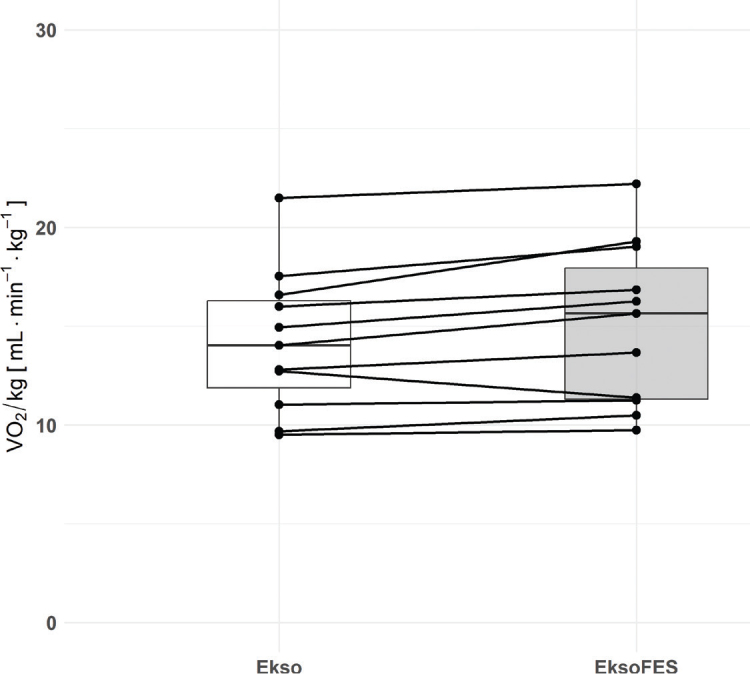
Comparison of V̇O_2_/kg (primary outcome) during the last 2 minof a 6-min walking test with the EksoNR exoskeleton with (EksoFES) and without (Ekso) gait-synchronized functional electrical stimulation. Linked data points are from the same subject.

In 10 of 11 participants, V̇O_2_/kg was higher in the EksoFES condition, resulting in a significant difference between conditions for our sample (β = 0.86, SE = 0.31, t (10) = 2.82, *p* = 0.02, Fig. 2). Notably, the test sequence had no significant effect (χ^2^(1) = 0.02, *p* = 0.88).

### Secondary outcomes

Secondary outcome measures are summarized in Table II. Independent of the sequence, V̇_E_, HR, and EE were significantly elevated in the EksoFES condition compared with Ekso without FES. For the rest of the parameters, no main effect of the condition was observed.

**Table II:** Secondary cardiorespiratory outcomes, distance covered, and rate of perceived exertion during 6-min walking tests with (EksoFES) and without (Ekso) functional electrical stimulation

Variable	Mean (SD)	Estimate, *β*	Standard error, SE	*t* (*β*/SE)	*p*-value
Minute ventilation, V̇_E_ (L/min)					
Ekso	31.96 (± 7.75)				
Ekso FES	34.69 (± 9.16)	2.74	1.02	2.70	0.02
Heart rate, HR (bpm)					
Ekso	95.84 (± 15.89)				
Ekso FES	98.53 (± 16.03)	2.68	1.06	2.52	0.03
Breathing frequency, BF (1/min)					
Ekso	28.68 (± 6.48)				
Ekso FES	28.67 (± 4.79)	–0.01	0.82	–0.01	1.00
Respiratory exchange ratio, RER					
Ekso	0.77 (± 0.05)				
Ekso FES	0.80 (± 0.06)	0.03	0.02	1.85	0.09
Energy expenditure, EE (Mets)					
Ekso	4.06 (± 1.03)				
Ekso FES	4.31 (± 1.17)	0.24	0.09	2.79	0.02
Distance (m)					
Ekso	127.3 (± 40.3)				
Ekso FES	130.5 (± 43.4)	3.18	3.04	1.05	0.32
Rate of perceived exertion, RPE (Borg 6–20)					
Ekso	11.45 (± 2.11)				
Ekso FES	11.27 (± 2.37)	–0.18	0.96	–0.19	0.85

bpm: beats per minute; SD: standard deviation, *t*: t-statistic from linear mixed-effect model.

### Correlation analysis

**Fig. 3:**
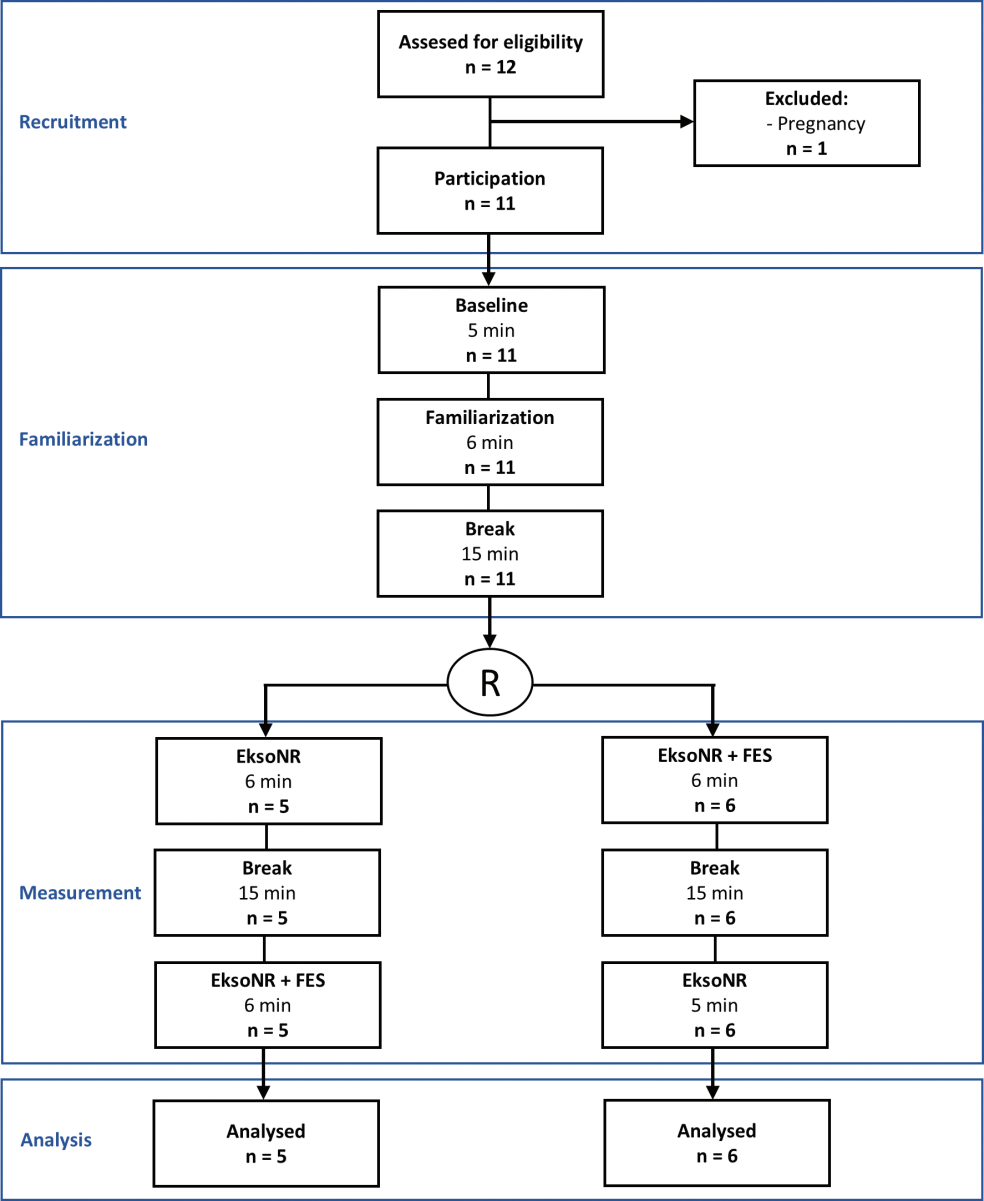
Correlation between the delta V̇O_2_/kg of the EksoFES compared with the Ekso alone condition and (A) mean stimulation amplitude of all stimulated muscles (i.e., gluteal muscles, hamstrings, quadriceps, and gastrocnemius), (B) mean distance covered during both 6-min walking tests, and (C) age.

Delta V̇O_2_/kg between conditions did not correlate with stimulation amplitude (*r* = 0.36, *p* = 0.27, Fig. 3A), average 6MWT distance (*r* = 0.44, *p* = 0.18, Fig. 3B) or age of the participants (*r* = –0.00, *p* = 0.99, Fig. 3C).

### Adverse events

No adverse events have occurred during this investigation. Two device deficiencies were reported: 1 electrode patch failure and 1 cable rupture, both while walking with FES. Importantly, all subjects could finish the investigation according to the study protocol.

## DISCUSSION

In this cross-sectional study with a convenience sample of 11 individuals with incomplete SCI, we employed a randomized crossover design to evaluate the additional cardiorespiratory demand of combining exoskeleton-assisted walking with FES. Participants performed 6MWTs with and without FES while using the EksoNR, allowing for a direct comparison of physiological responses between conditions. The findings indicate that integrating FES with exoskeleton-assisted walking increases cardiorespiratory demand by 6% from 14.21 to 15.07 mL·min^-^¹·kg^-^¹, supporting our primary hypothesis.

### Primary outcome

The increased cardiorespiratory demand may be attributed to the recruitment of additional muscle fibres through FES during exoskeleton-assisted ambulation, leading to a higher oxygen demand compared with walking with the EksoNR alone (23). While the addition of FES led to an overall increase in V̇O_2_/kg across the sample, interindividual variability was observed, with 1 of the 11 participants exhibiting a decrease (see Fig. 2). Multiple factors may contribute to the observed variability. Hypothetically, individuals with preserved gait function, a greater proportion of residual innervated muscles, and minimal subcutaneous adipose tissue may derive greater benefits from FES. In addition, partial damage to the lower motor neuron despite a good response to stimulation via nerve cannot be ruled out, especially by T10 lesions and below (30). Moreover, limb muscles in older adults are 25–35% smaller, contain significantly more fat and connective tissue compared with those in younger individuals (31, 32), and are thus less responsive to FES. However, our findings did not reveal a diminished FES response associated with age-related muscle mass loss (see Fig. 3C). This may be explained by the fact that, at any age, muscle atrophy following SCI is highly dependent on the lesion level and the extent of remaining muscle innervation (33). Hence, these factors have probably outweighed any age-related effects.

The negative changes experienced by 1 participant in the EksoFES condition compared with Ekso alone may be due to multiple factors. One possible explanation is the participant’s BMI of 31.9, indicating a considerable level of subcutaneous fat. This may have affected the effectiveness of FES, as higher fat mass can interfere with electrical stimulation (38). Another factor could be the participant’s limited remaining walking ability with the consequence that this was the only measurement conducted in Ekso’s maximal mode. In this mode, the exoskeleton provides substantially more support compared with the adaptive mode, making the walking movements more passive. This increased passivity may affect the additional impact of FES, potentially influencing neuromuscular activation. Moreover, as mentioned before, the participant initially struggled to adapt to the gait pattern imposed by the device, affecting performance in the first 6MWT with FES. In contrast, the second 6MWT (without FES) was smoother, as reflected in a 20% increase in distance walked (65 m vs 55 m). While the 6MWT distances were similar between conditions across the entire sample (see Table II), the improvement observed in this specific case could explain why physiological parameters were elevated during the non-FES condition. This highlights the importance of ensuring sufficient familiarization time to minimize learning effects that might occur during the actual measurements. However, it is important to mention that the randomized assignment of the sequence accounted for systematic order effects in the study design. Additionally, the sequence was appropriately considered in the statistical analysis and does not appear at all to contribute significantly to explaining our results. Hence, cardiorespiratory demand was higher in the EksoFES condition, regardless of whether it was performed first or second.

FES exerts its influence on cardiovascular function through both direct and indirect mechanisms, primarily by means of stimulation to the skeletal muscles. These, in turn, impact haemodynamics and autonomic regulation. When large muscle groups (e.g., quadriceps, gluteals) are stimulated via FES, venous return is enhanced, increasing stroke volume and cardiac output. Research has demonstrated that individuals who utilize FES cycling over an extended period of time exhibit an increase in peak oxygen uptake, comparable to the effects observed in able-bodied individuals following endurance training (34). Our findings demonstrate that the metabolic impact may extend beyond FES cycling, encompassing FES-assisted robot-assisted walking activities in individuals with incomplete SCI.

### Secondary outcomes

Among all outcome measures, the greatest within-subject difference was observed for V̇_E_ with an increase of about 8.5% due to FES. V̇_E_ is the product of the tidal volume and BF. While BF remained relatively unchanged, tidal volume was significantly higher in the EksoFES condition, thereby driving the overall increase in V̇_E_. This observation aligns with previous research indicating that with increasing physical demands individuals initially increase tidal volume but not BF to meet oxygen consumption needs (35). Similarly to V̇E, HR also showed a significant increase, albeit to a lesser extent of approximately 3%, further indicating that walking with FES was more demanding.

Obviously, energy expenditure increased at the same rate as V̇O_2_/kg, as it is directly derived from it. This finding is noteworthy, as higher energy expenditure corresponds to increased calorie consumption, which is particularly relevant in individuals with SCI. Given that weight management is a common challenge in this population, even a small rise in calorie consumption could potentially have a positive impact. Moreover, although the RER increased by approximately 4% in the EksoFES condition, this difference was not statistically significant. A higher RER suggests a greater reliance on carbohydrates for energy delivery, as they break down more quickly and require less oxygen for metabolism (36). However, the observed RER values (around 0.8) clearly indicate that walking with the exoskeleton, in both conditions, remained a submaximal effort and was likely within the moderate-intensity exercise range for most participants in our sample.

Finally, the distance covered in the 6MWT and RPE values was comparable between conditions. Distance was measured at 5-m intervals and rounded accordingly. Therefore, the additional 3 m covered in the EksoFES condition is negligible, allowing us to conclude that participants performed similarly in both conditions. RPE is a subjective parameter. Even though the EksoFES condition based on the physiological parameters was somewhat more physically demanding, this was apparently not perceived as such by the participants. After all, the difference was too small.

### Implications for practice

Taking cardiorespiratory and metabolic parameters into account, our observations are in line with the positive clinical experience of combining exoskeleton-assisted walking training with FES, if in accordance with the current therapy goals. Our findings indicate that the interindividual variability in the increase in cardiometabolic demand when combining exoskeleton-assisted walking with FES could not be attributed to higher stimulation amplitudes, age, or longer distances covered during the 6MWT with additional FES. Instead, other factors like the electrode placement, the remaining innervated muscle mass available for stimulation, the amount of subcutaneous fat mass, preconditioning for FES, and the potentially not sufficiently individualized stimulation parameters may have played a role (37–40). For everyday practice, however, it is important to note that combining exoskeleton training with FES also involves additional financial costs and time investment. These include expenses for the FES interface, stimulator, and electrodes, as well as the extra time needed to adjust FES parameters and apply or remove the electrodes before and after each training session. The results presented here provide justification for this effort and expenses; however, the results of the ongoing training study must be awaited before a final conclusion can be drawn.

### Limitations

As a convenience sample was used, the observed interindividual differences in V̇O_2_/kg changes may have been influenced by factors related to the recruitment process. Participants were exclusively selected from the Ekso-FES main project, meaning that some had recent training experience with Ekso and FES, while others had not used the device for a prolonged period. This heterogeneity may have affected familiarization and physiological responses. However, time since participation in the Ekso-FES trial did not significantly explain interindividual differences (*r* = –0.33, *p* = 0.35). Variations in training status are, nonetheless, inevitable and may also reflect real-world clinical conditions. Moreover, results may have been influenced by variability in chronicity (6.5 ± 6.9 years, see Table I), and the heterogeneity of lesion level indicates that different muscle groups were affected across participants. Although a more homogeneous cohort would have been preferable, such recruitment is challenging in clinical SCI studies and constrains generalizability. Measurements were obtained during a single visit, which may limit reproducibility. Multiple testing sessions would have provided more robust data but were not feasible in the present study. Furthermore, participants walked at a self-selected speed, which may have influenced the cardiorespiratory response. Unlike treadmill-based protocols, speed standardization is challenging in overground robotic gait due to individualized device settings; thus, self-selected speed is inherent to this type of assessment.

### Conclusion

This study demonstrates that the combination of FES and robotic-assisted walking increases cardiorespiratory demand in individuals with chronic incomplete SCI, making it a promising option to intensify exoskeleton-assisted gait training. The findings suggest that FES enhances muscle recruitment and metabolic activity, potentially contributing to more effective training sessions. Whether structured intensive training also results in a long-term improvement of gait function is currently being investigated. While the overall trends support the use of exoskeleton-assisted gait training combined with FES, the variability in the physiological responses highlights the need for further research.

## Data Availability

Data that support the findings of this study are available from the corresponding author upon reasonable request.

## References

[1] Perale G, Rossi F. Spinal cord injury (SCI) repair strategies. Sawston, UK: Woodhead Publishing; 2019.

[2] Hodgkiss DD, Bhangu GS, Lunny C, Jutzeler CR, Chiou SY, Walter M, et al. Exercise and aerobic capacity in individuals with spinal cord injury: a systematic review with meta-analysis and meta-regression. PLoS Med 2023; 20: e1004082. 10.1371/journal.pmed.1004082 38011304 PMC10712898

[3] Kirshblum S, Snider B, Eren F, Guest J. Characterizing natural recovery after traumatic spinal cord injury. J Neurotrauma 2021; 38: 1267–1284. 10.1089/neu.2020.7473 33339474 PMC8080912

[4] Kirshblum SC, Burns SP, Biering-Sorensen F, Donovan W, Graves DE, Jha A, et al. International standards for neurological classification of spinal cord injury (revised 2011). J Spinal Cord Med 2011; 34: 535–546. 10.1179/204577211X13207446293695 22330108 PMC3232636

[5] Sisto SA, Evans N. Activity and fitness in spinal cord injury: review and update. Curr Phys Med Rehabil Rep 2014; 2: 147–157. 10.1007/s40141-014-0057-y

[6] Karlsson AK. Autonomic dysfunction in spinal cord injury: clinical presentation of symptoms and signs. Prog Brain Res 2006; 152: 1–8. 10.1016/S0079-6123(05)52034-X 16198689

[7] Krueger H, Noonan VK, Trenaman LM, Joshi P, Rivers CS. The economic burden of traumatic spinal cord injury in Canada. Chronic Dis Inj Can 2013; 33: 113–122. 10.24095/hpcdp.33.3.01 23735450

[8] Behrman AL, Bowden MG, Nair PM. Neuroplasticity after spinal cord injury and training: an emerging paradigm shift in rehabilitation and walking recovery. Phys Ther 2006; 86: 1406–1425. 10.2522/ptj.20050212 17012645

[9] Bach Baunsgaard C, Vig Nissen U, Katrin Brust A, Frotzler A, Ribeill C, Kalke YB, et al. Gait training after spinal cord injury: safety, feasibility and gait function following 8 weeks of training with the exoskeletons from Ekso Bionics. Spinal Cord 2018; 56: 106–116. 10.1038/s41393-017-0013-7 29105657

[10] Nam KY, Kim HJ, Kwon BS, Park JW, Lee HJ, Yoo A. Robot-assisted gait training (Lokomat) improves walking function and activity in people with spinal cord injury: a systematic review. J Neuroeng Rehabil 2017; 14: 24. 10.1186/s12984-017-0232-3 28330471 PMC5363005

[11] Malik AN, Tariq H, Afridi A, Rathore FA. Technological advancements in stroke rehabilitation. J Pak Med Assoc 2022; 72: 1672-1674. 36280946 10.47391/JPMA.22-90

[12] Thomas C, Zaidner E, Calancie B, Broton J, Bigland-Ritchie B. Muscle weakness, paralysis, and atrophy after human cervical spinal cord injury. Exp Neurol 1997; 148: 414–423. 10.1006/exnr.1997.6690 9417821

[13] Bhatia D, Bansal G, Tewari R, Shukla K. State of art: functional electrical stimulation (FES). Int J Biomed Eng Technol 2011; 5: 77–99. 10.1504/IJBET.2011.038474

[14] Crameri RM, Cooper P, Sinclair PJ, Bryant G, Weston A. Effect of load during electrical stimulation training in spinal cord injury. Muscle Nerve 2004; 29: 104–111. 10.1002/mus.10522 14694505

[15] Davis GM, Hamzaid NA, Fornusek C. Cardiorespiratory, metabolic, and biomechanical responses during functional electrical stimulation leg exercise: health and fitness benefits. Artif Organs 2008; 32: 625–629. 10.1111/j.1525-1594.2008.00622.x 18782133

[16] Fujita N, Murakami S, Fujino H. The combined effect of electrical stimulation and high-load isometric contraction on protein degradation pathways in muscle atrophy induced by hindlimb unloading. J Biomed Biotechnol 2011; 2011: 401493. 10.1155/2011/401493 22007142 PMC3190189

[17] Gordon T, Tyreman N. Electrical stimulation to promote muscle and motor unit force and endurance after spinal cord injury. J Physiol 2023; 601: 1449–1466. 10.1113/JP283972 36815721

[18] Gorgey AS, Shepherd C. Skeletal muscle hypertrophy and decreased intramuscular fat after unilateral resistance training in spinal cord injury: case report. J Spinal Cord Med 2010; 33: 90–95. 10.1080/10790268.2010.11689681 20397451 PMC2853337

[19] Haapala SA, Faghri PD, Adams DJ. Leg joint power output during progressive resistance FES-LCE cycling in SCI subjects: developing an index of fatigue. J Neuroeng Rehabil 2008; 5: 14. 10.1186/1743-0003-5-14 18439300 PMC2396645

[20] Hasnan N, Ektas N, Tanhoffer AI, Tanhoffer R, Fornusek C, Middleton JW, et al. Exercise responses during functional electrical stimulation cycling in individuals with spinal cord injury. Med Sci Sports Exerc 2013; 45: 1131–1138. 10.1249/MSS.0b013e3182805d5a 23685444

[21] Hettinga DM, Andrews BJ. Oxygen consumption during functional electrical stimulation-assisted exercise in persons with spinal cord injury: implications for fitness and health. Sports Med 2008; 38: 825–838. 10.2165/00007256-200838100-00003 18803435

[22] Sabatier MJ, Stoner L, Mahoney ET, Black C, Elder C, Dudley GA, et al. Electrically stimulated resistance training in SCI individuals increases muscle fatigue resistance but not femoral artery size or blood flow. Spinal Cord 2006; 44: 227–233. 10.1038/sj.sc.3101834 16158074

[23] Ho CH, Triolo RJ, Elias AL, Kilgore KL, DiMarco AF, Bogie K, et al. Functional electrical stimulation and spinal cord injury. Phys Med Rehabil Clin N Am 2014; 25: 631. 10.1016/j.pmr.2014.05.001 25064792 PMC4519233

[24] Petrofsky JS, Phillips CA. The use of functional electrical stimulation for rehabilitation of spinal cord injured patients. Cent Nerv Syst Trauma 1984; 1: 57–74. 10.1089/cns.1984.1.57 6400201

[25] Tajali S, Iwasa SN, Sin V, Atputharaj S, Desai Kapadia N, Musselman KE, et al. The orthotic effects of different functional electrical stimulation protocols on walking performance in individuals with incomplete spinal cord injury: a case series. Top Spinal Cord Inj Rehabil 2023; 29: 142–152. 10.46292/sci23-00021S 38174132 PMC10759841

[26] Glasheen E, Kressler J, Domingo A. Oxygen consumption and substrate utilization during bionic ambulation with electrical stimulation for SCI. Arch Phys Med Rehabil 2018; 99: e209. 10.1016/j.apmr.2018.09.061

[27] Perry J, Burnfield JM. Gait analysis: normal and pathological function. Thorofare, NJ: SLACK Inc; 2010.

[28] Borg G. Borg’s perceived exertion and pain scales, Champaign, IL: Human Kinetics; 1998.

[29] Bates D, Mächler M, Bolker B, Walker S. Fitting linear mixed-effects models using lme4. J Stat Software 2015; 67: 1–48. 10.18637/jss.v067.i01

[30] Berger MJ, Adewuyi AA, Doherty C, Hanlan AK, Morin C, O’Connor R, et al. Segmental infralesional pathological spontaneous activity in subacute traumatic spinal cord injury. Muscle Nerve 2024; 69: 403–408. 10.1002/mus.28053 38294062

[31] Lexell J. Human aging, muscle mass, and fiber type composition. J Gerontol A Biol Sci Med Sci 1995; 50(Special issue): 11–16. 10.1093/gerona/50A.Special_Issue.11 7493202

[32] Booth FW, Weeden SH, Tseng BS. Effect of aging on human skeletal muscle and motor function. Med Sci Sports Exerc 1994; 26: 556–560. 10.1249/00005768-199405000-00006 8007802

[33] Gordon T, Mao J. Muscle atrophy and procedures for training after spinal cord injury. Phys Ther 1994; 74: 50–60. 10.1093/ptj/74.1.50 8265728

[34] Mate S, Corr N, Hackett D, Barnett M, Singh MF, Fornusek C. Functional electrical stimulation combined with voluntary cycling accentuates VO(2) response in people with severe multiple sclerosis: a pilot study. Mult Scler Relat Disord 2024; 85: 105552. 10.1016/j.msard.2024.105552 38537509

[35] Nicolò A, Sacchetti M. Differential control of respiratory frequency and tidal volume during exercise. Eur J Appl Physiol 2023; 123: 215–242. 10.1007/s00421-022-05077-0 36326866

[36] Melzer K. Carbohydrate and fat utilization during rest and physical activity. E Spen Eur E J Clin Nutr Metab 2011; 6: e45–e52. 10.1016/j.eclnm.2011.01.005

[37] Doheny EP, Caulfield BM, Minogue CM, Lowery MM. The effect of subcutaneous fat thickness on the efficacy of transcutaneous electrical stimulation. IEEE Eng Med Biol Soc Conf 2008: 5684–5687. 10.1109/IEMBS.2008.4650504 19164007

[38] Forrester BJ, Petrofsky JS. Effect of electrode size, shape, and placement during electrical stimulation. J Appl Res 2004; 4: 346–354.

[39] Kemp J, Després O, Pebayle T, Dufour A. Age-related decrease in sensitivity to electrical stimulation is unrelated to skin conductance: an evoked potentials study. Clin Neurophysiol 2014; 125: 602–607. 10.1016/j.clinph.2013.08.020 24070673

[40] van der Scheer JW, Goosey-Tolfrey VL, Valentino SE, Davis GM, Ho CH. Functional electrical stimulation cycling exercise after spinal cord injury: a systematic review of health and fitness-related outcomes. J Neuroeng Rehabil 2021; 18: 99. 10.1186/s12984-021-00882-8 34118958 PMC8196442

